# The Discrimination Power of Structural SuperIndices

**DOI:** 10.1371/journal.pone.0070551

**Published:** 2013-07-25

**Authors:** Matthias Dehmer, Abbe Mowshowitz

**Affiliations:** 1 Institute for Bioinformatics and Translational Research, Hall in Tyrol, Austria; 2 Department of Computer Science, The City College of New York, New York, New York, United States of America; Rensselaer Polytechnic Institute, United States of America

## Abstract

In this paper, we evaluate the discrimination power of structural superindices. Superindices for graphs represent measures composed of other structural indices. In particular, we compare the discrimination power of the superindices with those of individual graph descriptors. In addition, we perform a statistical analysis to generalize our findings to large graphs.

## Introduction

The absence of a polynomial time algorithm for determining if two arbitrary graphs are isomorphic has stimulated efforts to develop efficient heuristics that work in almost all cases. In particular, research on structural network measures has been undertaken in recent decades, see, e.g., [1]–[6]. Several different types of network measures have been developed. Some of them have been used to characterize the structure of graphs locally or globally [2]–[7]. Others have been used to characterize graphs quantitatively, and these have been applied to problems in areas such as structural chemistry, structural drug design, ecology, and computational physics [2], [8]–[10]. Bonchev [11] and Balaban et al. [12] developed structural indices to detect branching in molecular graphs. In addition to research directed at measuring structural features of a given network, work has been carried out on comparative network measures [13]–[16]. Examples include such work as graph similarity and graph distance measures which have been applied to graph clustering and other problems, see [17]–[19].

Properties of structural measures have also been examined in some detail. Research in this area encompasses investigations of the mathematical interrelations between network measures [20], [21], correlations between measures [22], [23], and their respective discrimination powers (also called *uniqueness*) [24]–[29]. Discrimination power (or the uniqueness property) is the central concern of this paper. In addition to earlier work on the uniqueness of structural graph measures [24]–[27], [29], [30], Dehmer et al. [28], [31] recently performed large scale analyses of the uniqueness of information-theoretic, degree-based and eigenvalue-based network measures. Here we focus on single indices defined relative to graph decompositions such as those induced by symmetry structure, distances, vertices, chromatic features, etc. Such an index is a mapping 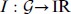 and can be interpreted as a graph complexity measure [2], [5], [9]. Single indices interpreted as graph invariants [6] have been studied in areas such as structural chemistry [3], [32] and computer science [33]. Also, we emphasize that approaches employing single indices for finding complete graph invariants have failed so far [32], [34], [35]. A complete graph invariant is an index that distinguishes between non-isomorphic graphs in a given collection. The reason for their failure is that every known single index has a certain degree of degeneracy [25], [35], that is, the measure can not distinguish non-isomorphic graphs by its values. Hence, single structural indices are not suitable for determining graph isomorphism, see [35].

In this paper, we explore the uniqueness of so-called *superindices* [25], [36], [37] for graphs (see section ‘SuperIndices’). Such superindices have been studied in structural chemistry and other disciplines [25], [36], [37]. A superindex is a composition of several structural index components, and is designed to obtain a measure which captures structural information more meaningfully than the individual components by themselves. To the best of our knowledge, the uniqueness of superindices [25] has not yet been explored to any great extent. To this end we use exhaustively generated general graphs [28] rather than any special graph classes such as chemical graphs [26], [29], [30]. The reason for using exhaustively generated general graphs (i.e., graphs without any structural constraints [28]) is to study the uniqueness of the superindices applied to arbitrary graphs. In short, the problem we address is the use of structural superindices that appear useful in determining graph isomorphism. Superindices are not restricted to any particular class of graphs - they can be applied to arbitrary graphs. Furthermore, a graph index is a measure that maps a single graph to the reals. In contrast, a graph metric [14], [38], [39] is a comparative measure designed to determine the structural similarity between graphs. Those metrics will not be used in this paper. Other graph measures such as the clustering coefficient or degree-based measures do not quantify structural features of graphs meaningfully as they exhibit a high degree of degeneracy [40].

## Methods

### SuperIndices

Superindices [25], [36], [37] are combinations of existing indices, where “combination” means algebraic or transcendental operations on the component indices. The term *superindex* was coined by Bonchev et al. [25] who devised superindices to achieve better discrimination between isomers than was possible using individual graph measures. Dehmer et al. [36] applied information-theoretic superindices to the Ames benchmark dataset of Hansen et al. [41] using supervised machine learning. In addition, Pogliani [37] derived certain superindices and demonstrated their power to predict melting points.

Let 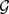 be a graph class and 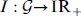 a topological index (or descriptor). Given 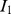 and 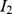 we define the following superindices, chosen because they are the simplest and most obvious linear combinations of two indices, and turn out to have high discrimination power, and, after all, this is the acid test of the utility of the indices. It is of course possible that other combination methods, based for example on rank reduction techniques such as Singular Value Decomposition, would produce indices with even greater discrimination power. However, that is something to be explored in future papers. We define:
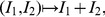
(1)

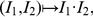
(2)

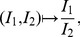
(3)

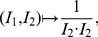
(4)

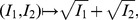
(5)

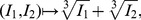
(6)

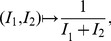
(7)

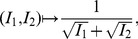
(8)

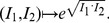
(9)


Balaban et al. [12] proposed similar superindices in QSAR/QSPR [12], [42]. That selection proved quite useful and has influenced our choice of superindices for the current study of uniqueness. In the following sections, we analyze the discrimination power of these superindices numerically and statistically. In particular, we demonstrate that some superindices far outperform the underlying single descriptors.

### Data and Computation

The uniqueness of the superindices listed above has been analyzed on a collection of exhaustively generated graphs [28]. This collection, denoted 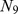 (with 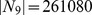) [28], consists of all non-isomorphic connected graphs on 9 vertices. As in [28], the graphs in this collection were generated by the program geng from the Nauty package [43]. The individual as well as the superindices were calculated with the aid to the R-package QuACN [44], [45].

The random graph construction model was selected because it yields the most general class of graphs, and seems appropriate for an initial study of the discrimination power of superindices. Other construction methods, e.g., [46] are also of interest, especially because they model many real world graphs known to exhibit a power law distribution. However, application of the superindices to graphs produced by other construction methods is beyond the scope of the current paper.

## Results

### Numerical Results

Table 1 presents the QuACN-descriptors [44] with their input options (parameter) and their abbreviations. Superindices with components drawn from the descriptors in Table 1 have been calculated. The results of these computations (discussed below) are shown by Tables 5, 6, 7, 8, 9, 10, 11, 12. Table 4 shows the uniqueness of QuACN-descriptors for given ndv-values, i.e., the number of the non-distinguishable values (graphs) for a particular index and sensitivity
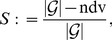
(10)see [26], [28]. The tables show that only a few of the QuACN-descriptors possess high uniqueness, having 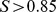. Examples of such highly discriminating indices are infotheolin2, infotheoquad2, infotheoexp2, infotheoexp3, laplacianEstrada, minBalabanID, eigenvalaugement, eigenvalextadj, eigenvalvertconnect, eigenvalrandomwalk, eigenvalweightedlin, eigenvalweightedexp. High discrimination power has already been observed (see [28], [31]) for some of the indices, namely, information-theoretic measures (e.g., infotheolin2, infotheoquad2, infotheoexp2 etc.) and the entropic eigenvalue-based measures (eigenvalaugement, eigenvalextadj, eigenvalvertconnect etc.) due to Dehmer [47], [48]. Note that the uniqueness of the minBalabanID [49] is less than the uniqueness of some of the above mentioned measures due to Dehmer [28], [31]. Most of the so-called molecular ID numbers (such as minBalabanID) appear to be highly discriminating but have never been evaluated on general graph classes such as exhaustively generated general graphs. It has also been observed that the uniqueness of structural graph indices depend on the graph class under consideration, see [28], [31], [50].

**Table 1:** Descriptors from QuACN [44] where g denotes an input graph.

QuACN-descriptors with input options	Abbreviation
augmentedZagreb(g)	augmentedZagreb
balabanJ(g)	balabanJ
balabanlike1(g)	balabanlike1
balabanlike2(g)	balabanlike2
bertz(g)	bertz
bonchev1(g)	bonchev1
bonchev2(g)	bonchev2
bonchev3(g)	bonchev3
compactness(g)	compactness
complexityIndexB(g)	complexityIndexB
randic(g)	randic
wiener(g)	wiener
zagreb1(g)	zagreb1
zagreb2(g)	zagreb2
harary(g)	harary
normalizedEdgeComplexity(g)	normalizedEdgeComplexity
radialCentric(g)	radialCentric
infoTheoreticGCM(g,coeff = “lin”,infofunct = “sphere”,lambda = 1000)	infotheolin1
infoTheoreticGCM(g,coeff = “lin”,infofunct = “vertcent”,lambda = 1000)	infotheolin2
infoTheoreticGCM(g,coeff = “quad”,infofunct = “sphere”,lambda = 1000)	infotheoquad1
infoTheoreticGCM(g,coeff = “quad”,infofunct = “vertcent”,lambda = 1000)	infotheoquad2
infoTheoreticGCM(g,coeff = “exp”,infofunct = “sphere”,lambda = 1000)	infotheoexp1
infoTheoreticGCM(g,coeff = “exp”,infofunct = “vertcent”,lambda = 1000)	infotheoexp2
infoTheoreticGCM(g,coeff = “exp”,infofunct = “degree”,lambda = 1000)	infotheoexp3
meanDistanceDeviation(g)	meanDistanceDeviation
productOfRowSums(g,log = T)	productofrowsums
hyperDistancePathIndex(g)	hyperDistancePathIndex
topologicalInfoContent(g)	topologicalinfocontent
vertexDegree(g)	vertexDegree
graphVertexComplexity(g)	graphVertexComplexity
graphIndexComplexity(g)	graphIndexComplexity
graphDistanceComplexity(g)	graphDistanceComplexity
informationLayerIndex(g)	informationLayerIndex
modifiedZagreb(g)	modifiedZagreb
minConnectivityID(g)	minConnectivityID
laplacianEnergy(g)	laplacianEnergy
laplacianEstrada(g)	laplacianEstrada
mediumArticulation(g)	mediumArticulation
minBalabanID(g)	minBalabanID
minConnectivityID(g)	minConnectivityID
modifiedZagreb(g)	modifiedZagreb
narumiKatayama(g)	narumiKatayama
offdiagonal(g)	offdiagonal
spanningTreeSensitivity(g)	spanningTreeSensitivity
spectralRadius(g)	spectralRadius
symmetryIndex(g)	symmetryIndex
variableZagreb(g)	variableZagreb
eigenvalueBased(g,adjacencyMatrix,1)	eigenvaladj
eigenvalueBased(g,laplaceMatrix,1)	eigenvallaplace
eigenvalueBased(g,distanceMatrix,1)	eigenvaldistance
eigenvalueBased(g,distancePathMatrix,1)	eigenvaldistancepath
eigenvalueBased(g,augmentedMatrix,1)	eigenvalaugement
eigenvalueBased(g,extendedAdjacencyMatrix,1)	eigenvalextadj
eigenvalueBased(g,vertConnectMatrix,1)	eigenvalvertconnect
eigenvalueBased(g,randomWalkMatrix,1)	eigenvalrandomwalk
eigenvalueBased(g,weightStrucFuncMatrix_lin,1)	eigenvalweightedlin
eigenvalueBased(g,weightStrucFuncMatrix_exp,1)	eigenvalweightedexp
infoTheoreticGCM(g,coeff = “lin”,infofunct = “pathlength”,lambda = 1000)	infotheolin3
infoTheoreticGCM(g,coeff = “quad”,infofunct = “pathlength”,lambda = 1000)	infotheoquad3
infoTheoreticGCM(g,coeff = “exp”,infofunct = “pathlength”,lambda = 1000)	infotheoexp4

**Table 4:** ndv-values for the individual QuACN-descriptors of graphs in 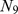.

Descriptors (abbreviation)	ndv	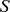
augmentedZagreb	241777	0.07394
balabanJ	156674	0.39990
balabanlike1	148132	0.43262
balabanlike2	148132	0.43262
bertz	261080	0.00000
bonchev1	260971	0.00042
bonchev2	260803	0.00106
bonchev3	260971	0.00042
compactness	261072	0.00003
complexityIndexB	237199	0.09147
randic	243413	0.06767
wiener	261072	0.00003
zagreb1	261078	0.00001
zagreb2	260931	0.00057
harary	261018	0.00024
normalizedEdgeComplexity	261078	0.00001
radialCentric	261079	0.00000
infotheolin1	249439	0.04459
infotheolin2	36310	0.86092
infotheoquad1	235044	0.09972
infotheoquad2	27032	0.89646
infotheoexp1	235055	0.09968
infotheoexp2	27017	0.89652
infotheoexp3	1877	0.99281
meanDistanceDeviation	261067	0.00005
productofrowsums	252262	0.03378
hyperDistancePathIndex	261054	0.00010
topologicalinfocontent	261080	0.00000
vertexDegree	261079	0.00000
graphVertexComplexity	260648	0.00165
graphIndexComplexity	44652	0.82897
graphDistanceComplexity	235233	0.09900
minConnectivityID	19842	0.92400
laplacianEnergy	59542	0.77194
laplacianEstrada	23393	0.91040
mediumArticulation	260576	0.00193
minBalabanID	18341	0.92975
modifiedZagreb	258293	0.01067
narumiKatayama	260925	0.00059
offdiagonal	259967	0.00426
spanningTreeSensitivity	44389	0.82998
spectralRadius	48120	0.81569
symmetryIndex	261070	0.00004
variableZagreb	258286	0.01070
eigenvaladj	42347	0.83780
eigenvallaplace	35206	0.86515
eigenvaldistance	23202	0.91113
eigenvaldistancepath	19982	0.92346
eigenvalaugement	0	1.00000
eigenvalextadj	479	0.99817
eigenvalvertconnect	1089	0.99583
eigenvalrandomwalk	1176	0.99550
eigenvalweightedlin	3693	0.98585
eigenvalweightedexp	4402	0.98314
infotheolin3	158391	0.39332
infotheoquad3	58196	0.77710
infotheoexp4	27017	0.89652

**Table 5:** ndv-values of graphs in 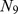 for different combinations of QuACN-descriptors from the first set of superindices.

Descriptors 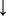 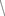 ndv 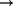	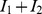	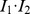	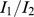	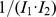	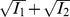
augmentedZagreb_eigenvalaugement	0	0	0	0	0
balabanJ_eigenvalaugement	0	0	0	0	0
balabanlike1_eigenvalaugement	0	0	0	0	0
balabanlike2_eigenvalaugement	0	0	0	0	0
bertz_eigenvalaugement	0	0	0	0	0
bonchev1_eigenvalaugement	0	0	0	0	0
bonchev2_eigenvalaugement	0	0	0	0	0
bonchev3_eigenvalaugement	0	0	0	0	0
compactness_eigenvalaugement	0	0	0	0	0
complexityIndexB_eigenvalaugement	0	0	0	0	0
randic_eigenvalaugement	0	0	0	0	0
wiener_eigenvalaugement	0	0	0	0	0
zagreb1_eigenvalaugement	0	0	0	0	0
zagreb2_eigenvalaugement	0	0	0	0	0
harary_eigenvalaugement	0	0	0	0	0
normalizedEdgeComplexity_eigenvalaugement	0	0	0	0	0
radialCentric_eigenvalaugement	0	79673	79673	79673	0
infotheolin1_eigenvalaugement	0	0	0	0	0
infotheolin2_eigenvalaugement	0	0	0	0	0
infotheolin2_eigenvalextadj	0	0	0	0	0
infotheoquad1_eigenvalaugement	0	0	0	0	0
infotheoquad2_eigenvalaugement	0	0	0	0	0
infotheoquad2_eigenvalextadj	0	0	0	0	0
infotheoexp1_eigenvalaugement	0	0	0	0	0
infotheoexp2_eigenvalaugement	0	0	0	0	0
infotheoexp2_eigenvalextadj	0	0	0	0	0
infotheoexp2_eigenvalvertconnect	0	0	0	2	2
infotheoexp2_eigenvalrandomwalk	0	0	2	2	2
infotheoexp3_laplacianEstrada	0	0	0	0	0
infotheoexp3_eigenvallaplace	0	0	0	0	0
infotheoexp3_eigenvaldistance	0	0	0	0	0
infotheoexp3_eigenvaldistancepath	0	0	0	0	0
infotheoexp3_eigenvalaugement	0	0	0	0	0
meanDistanceDeviation_eigenvalaugement	0	23	23	23	0
hyperDistancePathIndex_eigenvalaugement	0	0	0	0	0

**Table 6:** ndv-values of graphs in 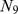 for different combinations of QuACN-descriptors from the second subset of superindices.

Descriptors 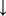 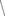 ndv 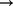	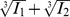	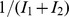	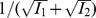	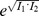
augmentedZagreb_eigenvalaugement	0	0	0	0
balabanJ_eigenvalaugement	0	0	0	0
balabanlike1_eigenvalaugement	0	0	0	0
balabanlike2_eigenvalaugement	0	0	0	0
bertz_eigenvalaugement	0	0	0	0
bonchev1_eigenvalaugement	0	0	0	0
bonchev2_eigenvalaugement	0	0	0	0
bonchev3_eigenvalaugement	0	0	0	0
compactness_eigenvalaugement	0	0	0	0
complexityIndexB_eigenvalaugement	0	0	0	0
randic_eigenvalaugement	0	0	0	0
wiener_eigenvalaugement	0	0	0	0
zagreb1_eigenvalaugement	0	0	0	0
zagreb2_eigenvalaugement	0	0	0	0
harary_eigenvalaugement	0	0	0	0
normalizedEdgeComplexity_eigenvalaugement	0	0	0	0
radialCentric_eigenvalaugement	0	0	0	79673
infotheolin1_eigenvalaugement	0	0	0	0
infotheolin2_eigenvalaugement	0	0	0	0
infotheolin2_eigenvalextadj	0	0	0	0
infotheoquad1_eigenvalaugement	0	0	0	0
infotheoquad2_eigenvalaugement	0	0	0	0
infotheoquad2_eigenvalextadj	0	0	0	0
infotheoexp1_eigenvalaugement	0	0	0	0
infotheoexp2_eigenvalaugement	0	0	0	0
infotheoexp2_eigenvalextadj	0	0	0	0
infotheoexp2_eigenvalvertconnect	2	2	2	2
infotheoexp2_eigenvalrandomwalk	2	2	2	2
infotheoexp3_laplacianEstrada	0	0	0	0
infotheoexp3_eigenvallaplace	0	0	0	0
infotheoexp3_eigenvaldistance	0	0	0	0
infotheoexp3_eigenvaldistancepath	0	0	0	0
infotheoexp3_eigenvalaugement	0	0	0	0
meanDistanceDeviation_eigenvalaugement	0	0	0	23
hyperDistancePathIndex_eigenvalaugement	0	0	0	0

**Table 7:** ndv-values of graphs in 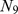 for different combinations of QuACN-descriptors from the first set of superindices (continued).

Descriptors 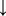 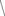 ndv 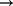	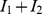	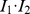	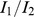	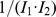	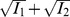
topologicalinfocontent_eigenvalaugement	0	22	22	22	0
vertexDegree_eigenvalaugement	0	22	22	22	0
graphVertexComplexity_eigenvalaugement	0	0	0	0	0
graphIndexComplexity_eigenvalaugement	0	0	0	0	0
graphDistanceComplexity_eigenvalaugement	0	0	0	0	0
minConnectivityID_eigenvalaugement	0	0	0	0	0
laplacianEnergy_eigenvalaugement	0	0	0	0	0
laplacianEstrada_eigenvalaugement	0	0	0	0	0
laplacianEstrada_eigenvalextadj	0	0	0	0	0
mediumArticulation_eigenvalaugement	0	2	2	2	0
minBalabanID_spanningTreeSensitivity	0	57	57	57	0
minBalabanID_eigenvalaugement	0	0	0	0	0
minBalabanID_eigenvalextadj	0	0	0	0	0
minBalabanID_eigenvalvertconnect	0	0	0	0	0
minBalabanID_eigenvalrandomwalk	0	0	0	0	0
modifiedZagreb_eigenvalaugement	0	0	0	0	0
offdiagonal_eigenvalaugement	0	30	30	30	0
spanningTreeSensitivity_eigenvalaugement	0	57	57	57	0
spectralRadius_eigenvalaugement	0	0	0	0	0
symmetryIndex_eigenvalaugement	0	70829	70829	70829	0
variableZagreb_eigenvalaugement	0	0	0	0	0
eigenvaladj_eigenvalaugement	0	0	0	0	0
eigenvallaplace_eigenvalaugement	0	0	0	0	0
eigenvallaplace_eigenvalextadj	0	0	0	0	0
eigenvaldistance_eigenvalaugement	0	0	0	0	0
eigenvaldistancepath_eigenvalaugement	0	0	0	0	0
eigenvalaugement_eigenvalextadj	0	0	0	0	0
eigenvalaugement_eigenvalvertconnect	0	0	0	0	0
eigenvalaugement_eigenvalrandomwalk	0	0	0	0	0
eigenvalaugement_eigenvalweightedlin	0	0	0	0	0
eigenvalaugement_eigenvalweightedexp	0	0	0	0	0
eigenvalaugement_infotheolin3	0	0	0	0	0
eigenvalaugement_infotheoquad3	0	0	0	0	0
eigenvalaugement_infotheoexp4	0	0	0	0	0
meanDistanceDeviation_eigenvalextadj	205	226	220	213	213

**Table 8:** ndv-values of graphs in 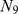 for different combinations of QuACN-descriptors from the second subset of superindices (continued).

Descriptors 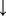 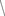 ndv 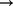	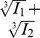	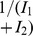	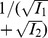	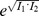
topologicalinfocontent_eigenvalaugement	0	0	0	22
vertexDegree_eigenvalaugement	0	0	0	22
graphVertexComplexity_eigenvalaugement	0	0	0	0
graphIndexComplexity_eigenvalaugement	0	0	0	0
graphDistanceComplexity_eigenvalaugement	0	0	0	0
minConnectivityID_eigenvalaugement	0	0	0	0
laplacianEnergy_eigenvalaugement	0	0	0	0
laplacianEstrada_eigenvalaugement	0	0	0	0
laplacianEstrada_eigenvalextadj	0	0	0	0
mediumArticulation_eigenvalaugement	0	0	0	2
minBalabanID_spanningTreeSensitivity	0	0	0	57
minBalabanID_eigenvalaugement	0	0	0	0
minBalabanID_eigenvalextadj	0	0	0	0
minBalabanID_eigenvalvertconnect	0	0	0	0
minBalabanID_eigenvalrandomwalk	0	0	0	0
modifiedZagreb_eigenvalaugement	0	0	0	0
offdiagonal_eigenvalaugement	0	0	0	30
spanningTreeSensitivity_eigenvalaugement	0	0	0	57
spectralRadius_eigenvalaugement	0	0	0	0
symmetryIndex_eigenvalaugement	0	0	0	70829
variableZagreb_eigenvalaugement	0	0	0	0
eigenvaladj_eigenvalaugement	0	0	0	0
eigenvallaplace_eigenvalaugement	0	0	0	0
eigenvallaplace_eigenvalextadj	0	0	0	0
eigenvaldistance_eigenvalaugement	0	0	0	0
eigenvaldistancepath_eigenvalaugement	0	0	0	0
eigenvalaugement_eigenvalextadj	0	0	0	0
eigenvalaugement_eigenvalvertconnect	0	0	0	0
eigenvalaugement_eigenvalrandomwalk	0	0	0	0
eigenvalaugement_eigenvalweightedlin	0	0	0	0
eigenvalaugement_eigenvalweightedexp	0	0	0	0
eigenvalaugement_infotheolin3	0	0	0	0
eigenvalaugement_infotheoquad3	0	0	0	0
eigenvalaugement_infotheoexp4	0	0	0	0
meanDistanceDeviation_eigenvalextadj	219	199	215	216

**Table 9:** ndv-values of graphs in 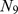 for different combinations of QuACN-descriptors from the first subset of superindices (continued).

Descriptors 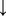 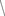 ndv 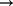	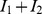	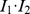	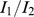	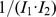	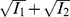
balabanlike1_spanningTreeSensitivity	208	161	215	221	200
infotheolin2_spanningTreeSensitivity	212	203	181	167	220
balabanJ_spanningTreeSensitivity	214	241	201	177	214
hyperDistancePathIndex_eigenvalextadj	215	201	207	202	215
radialCentric_eigenvalextadj	216	79793	79789	79779	232
spanningTreeSensitivity_infotheoquad3	216	219	213	171	214
balabanlike1_eigenvalextadj	217	201	197	201	211
wiener_eigenvalextadj	217	213	205	185	213
harary_eigenvalextadj	217	187	199	215	217
bonchev2_eigenvalextadj	219	191	170	209	217
symmetryIndex_eigenvalextadj	221	71031	71050	71040	225
eigenvaldistance_infotheoexp4	230	210	202	248	250
balabanlike2_eigenvalvertconnect	235	245	239	240	257
infotheoexp2_eigenvaldistance	238	190	248	246	248
infotheolin2_eigenvaldistancepath	240	220	246	248	250
infotheoquad2_eigenvaldistance	242	198	240	238	246
eigenvaldistancepath_infotheoexp4	242	208	218	250	252
infotheolin2_eigenvaldistance	244	216	246	238	244
infotheoexp2_eigenvaldistancepath	244	232	246	252	252
infotheoquad2_eigenvaldistancepath	246	212	248	246	250
balabanJ_eigenvalvertconnect	247	221	231	253	263
balabanlike2_eigenvalrandomwalk	247	249	245	245	259
balabanJ_eigenvalrandomwalk	255	229	239	261	261
balabanlike1_eigenvalvertconnect	261	245	237	245	247
eigenvaladj_infotheoexp4	262	226	222	286	288
balabanlike1_eigenvalrandomwalk	263	253	249	247	257
infotheolin2_eigenvaladj	264	212	280	282	286
infotheoexp2_eigenvaladj	264	216	282	280	288
infotheoquad2_eigenvaladj	268	216	284	288	288
infotheolin1_eigenvalvertconnect	273	241	305	297	309
infotheoquad1_eigenvalvertconnect	275	245	305	303	303
complexityIndexB_eigenvalvertconnect	287	259	295	289	305
graphDistanceComplexity_eigenvalvertconnect	287	245	299	303	311
narumiKatayama_eigenvalextadj	519	469	457	463	519
graphIndexComplexity_infotheoquad3	535	515	505	501	537

**Table 10:** ndv-values of graphs in 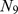 for different combinations of QuACN-descriptors from the second subset of superindices (continued).

Descriptors 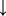 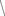 ndv 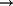	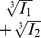	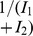	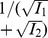	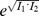
balabanlike1_spanningTreeSensitivity	200	192	200	217
infotheolin2_spanningTreeSensitivity	220	218	232	187
balabanJ_spanningTreeSensitivity	228	214	214	239
hyperDistancePathIndex_eigenvalextadj	219	219	219	146
radialCentric_eigenvalextadj	234	215	220	79793
spanningTreeSensitivity_cinfotheoquad3	230	228	226	205
balabanlike1_eigenvalextadj	215	211	217	187
wiener_eigenvalextadj	219	219	219	159
harary_eigenvalextadj	219	219	219	152
bonchev2_eigenvalextadj	217	219	217	132
symmetryIndex_eigenvalextadj	231	225	229	71036
eigenvaldistance_infotheoexp4	246	244	246	234
balabanlike2_eigenvalvertconnect	261	247	257	235
infotheoexp2_eigenvaldistance	250	248	250	246
infotheolin2_eigenvaldistancepath	248	250	248	246
infotheoquad2_eigenvaldistance	250	250	248	242
eigenvaldistancepath_infotheoexp4	252	246	252	248
infotheolin2_eigenvaldistance	248	246	252	240
infotheoexp2_eigenvaldistancepath	250	250	250	244
infotheoquad2_eigenvaldistancepath	250	248	252	244
balabanJ_eigenvalvertconnect	261	253	259	249
balabanlike2_eigenvalrandomwalk	259	255	263	235
balabanJ_eigenvalrandomwalk	259	253	263	257
balabanlike1_eigenvalvertconnect	263	253	263	247
eigenvaladj_infotheoexp4	294	286	288	270
balabanlike1_eigenvalrandomwalk	261	255	263	239
infotheolin2_eigenvaladj	292	284	292	272
infotheoexp2_eigenvaladj	294	284	286	274
infotheoquad2_eigenvaladj	290	282	292	272
infotheolin1_eigenvalvertconnect	313	307	307	293
infotheoquad1_eigenvalvertconnect	311	305	305	293
complexityIndexB_eigenvalvertconnect	309	307	311	293
graphDistanceComplexity_eigenvalvertconnect	307	309	305	295
narumiKatayama_eigenvalextadj	515	519	517	124837
graphIndexComplexity_infotheoquad3	545	537	539	481

**Table 11:** ndv-values of graphs in 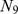 for different combinations of QuACN-descriptors from the first subset of superindices (continued).

Descriptors 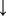 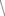 ndv 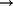	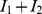	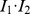	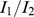	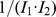	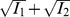
radialCentric_eigenvalvertconnect	551	80113	80081	80088	596
infotheoexp3_graphIndexComplexity	568	549	548	515	577
radialCentric_eigenvalrandomwalk	597	80160	80154	80130	615
topologicalinfocontent_eigenvalvertconnect	728	677	713	804	807
offdiagonal_eigenvalvertconnect	788	815	832	750	845
randic_eigenvalvertconnect	791	825	821	657	839
balabanlike2_laplacianEstrada	800	885	1102	1067	1015
randic_eigenvalrandomwalk	801	839	833	735	851
topologicalinfocontent_eigenvalrandomwalk	807	753	777	822	833
infotheoexp3_symmetryIndex	812	71572	71549	71565	811
balabanlike1_laplacianEstrada	820	861	1102	1055	1026
offdiagonal_eigenvalrandomwalk	831	849	854	812	857
bertz_eigenvalvertconnect	835	783	780	642	819
vertexDegree_eigenvalvertconnect	835	810	761	899	948
mediumArticulation_eigenvalvertconnect	841	841	820	791	887
bertz_eigenvalrandomwalk	845	811	818	748	839
balabanJ_laplacianEstrada	849	1051	714	841	1030
mediumArticulation_eigenvalrandomwalk	865	889	860	839	907
zagreb2_eigenvalvertconnect	869	747	743	785	867
zagreb2_eigenvalrandomwalk	869	795	823	819	867
augmentedZagreb_eigenvalvertconnect	870	752	742	769	866
augmentedZagreb_eigenvalrandomwalk	870	792	819	821	870
laplacianEstrada_spanningTreeSensitivity	883	836	812	805	1027
infotheoexp2_infotheoexp3	887	858	889	881	916
infotheoquad2_infotheoexp3	892	857	877	877	919
infotheolin2_infotheoexp3	901	844	875	875	913
infotheoexp3_infotheoquad3	901	852	845	883	917
infotheoexp3_infotheoexp4	902	827	866	870	907
infotheoexp3_infotheolin3	907	836	839	879	908
vertexDegree_eigenvalrandomwalk	907	896	891	953	956
narumiKatayama_eigenvalvertconnect	917	790	795	777	917
narumiKatayama_eigenvalrandomwalk	917	852	857	846	917
infotheoexp3_minBalabanID	949	889	910	846	939

**Table 12:** ndv-values of graphs in 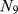 for different combinations of QuACN-descriptors from the second subset of superindices (continued).

Descriptors 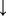 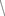 ndv 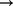	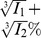	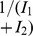	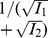	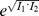
radialCentric_eigenvalvertconnect	604	562	575	80087
infotheoexp3_graphIndexComplexity	573	561	566	556
radialCentric_eigenvalrandomwalk	628	603	607	80150
topologicalinfocontent_eigenvalvertconnect	831	815	814	732
offdiagonal_eigenvalvertconnect	851	782	819	814
randic_eigenvalvertconnect	839	839	839	729
balabanlike2_laplacianEstrada	1596	1033	1495	112
randic_eigenvalrandomwalk	857	849	857	781
topologicalinfocontent_eigenvalrandomwalk	835	825	827	776
infotheoexp3_symmetryIndex	820	777	796	71592
balabanlike1_laplacianEstrada	1613	1077	1479	146
offdiagonal_eigenvalrandomwalk	859	817	835	846
bertz_eigenvalvertconnect	837	843	841	513
vertexDegree_eigenvalvertconnect	946	918	940	762
mediumArticulation_eigenvalvertconnect	899	843	875	810
bertz_eigenvalrandomwalk	839	845	845	612
balabanJ_laplacianEstrada	1639	1076	1465	102
mediumArticulation_eigenvalrandomwalk	905	893	883	864
zagreb2_eigenvalvertconnect	859	869	863	500
zagreb2_eigenvalrandomwalk	863	869	867	505
augmentedZagreb_eigenvalvertconnect	842	868	868	471
augmentedZagreb_eigenvalrandomwalk	862	870	868	522
laplacianEstrada_spanningTreeSensitivity	1244	767	1146	205
infotheoexp2_infotheoexp3	925	907	915	827
infotheoquad2_infotheoexp3	915	909	913	821
infotheolin2_infotheoexp3	915	920	912	834
infotheoexp3_infotheoquad3	924	922	915	821
infotheoexp3_infotheoexp4	917	916	915	811
infotheoexp3_infotheolin3	917	905	914	830
vertexDegree_eigenvalrandomwalk	956	932	938	863
narumiKatayama_eigenvalvertconnect	917	919	917	125640
narumiKatayama_eigenvalrandomwalk	917	919	917	125677
infotheoexp3_minBalabanID	942	930	945	824

Tables 5, 6, 7, 8 present the uniqueness results for certain combinations of descriptors involving the superindices. Each pair of tables shows the the results for two subsets of such indices. The first subset consists of Equations 1–5 (e.g., Table 5) and the second subset consists of Equations 6–9 (e.g., Table 6), respectively. For instance if we look at Table 5, we see that most of the superindices now discriminate the graphs perfectly (ndv = 0) even when indices with very low uniqueness (such as augmentedZagreb, bertz, wiener etc.) are involved. When applying the descriptors radialCentric and eigenvalaugement to the Equations representing the superindices, some of them are much less discriminating (ndv = 79676 corresponds to 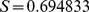). This is due to the fact that radialCentric has little discrimination power (it discriminate only two graphs out of 261080). A similar effect can be seen in Tables 9, 10, 11, 12. For instance, Table 9 shows that the composition (based on the superindices) of a descriptor with little discrimination power (e.g., narumiKatayama; ndv = 260925, 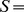 0.00059, see Table 4) with another descriptor having high discrimination power (e.g., eigenvalvertconnect; ndv = 1089, 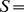 0.99583, see Table 4) leads again to a highly unique measure. In this particular case and by using the superindex 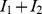, we find its discrimination power to be ndv = 535 and 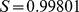. Uniqueness (measured by ndv and 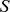) of the new measure is better than the uniqueness of the component measures, see Table 9. More extreme cases can be found in Table 12 defined as the composition of the two descriptors topologicalinfocontent and eigenvalvertconnect using the superindex 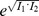. In short, Tables 5, 6, 7, 8, 9, 10, 11, 12 demonstrate that most of the superindices possess high uniqueness when one of the constituent graph measures has little discrimination power.

To better understand the behavior of these indices it would be desirable to explore the *structural interpretation* of these measures. Many of the constituent measures have a structural interpretation associated with a branching index [11], [22] (e.g., the Wiener index (wiener) or as a cyclicity index [12] (e.g., the Balaban index (balabanJ). A correlation analysis might be used to determine classes of superindices having a distinctive interpretation, e.g., branching, cyclicity, irregularity etc. Such an analysis would involve finding the correlations between 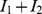 and 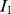, 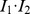 and 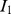, 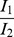 and 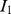, etc. However, this is beyond the scope of the present paper.

### Statistical Analysis

To determine the scalability of our findings on discrimination power of superindices applied to the graphs in 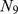, we have performed a statistical analysis. The aim of this analysis is to determine whether or not the results for determining uniqueness are statistically stable for graphs with larger numbers of vertices. Central to this analysis is a method for generating random graphs. We used *Bootstrapping* [51], [52] to estimate the underlying sampling distribution.

Let 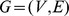 be a graph with 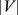 vertices and 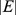 edges. Now, the size 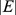 of the edge set of a connected random graph with 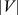 vertices satisfies.
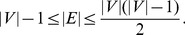
(11)


**Figure 1:**
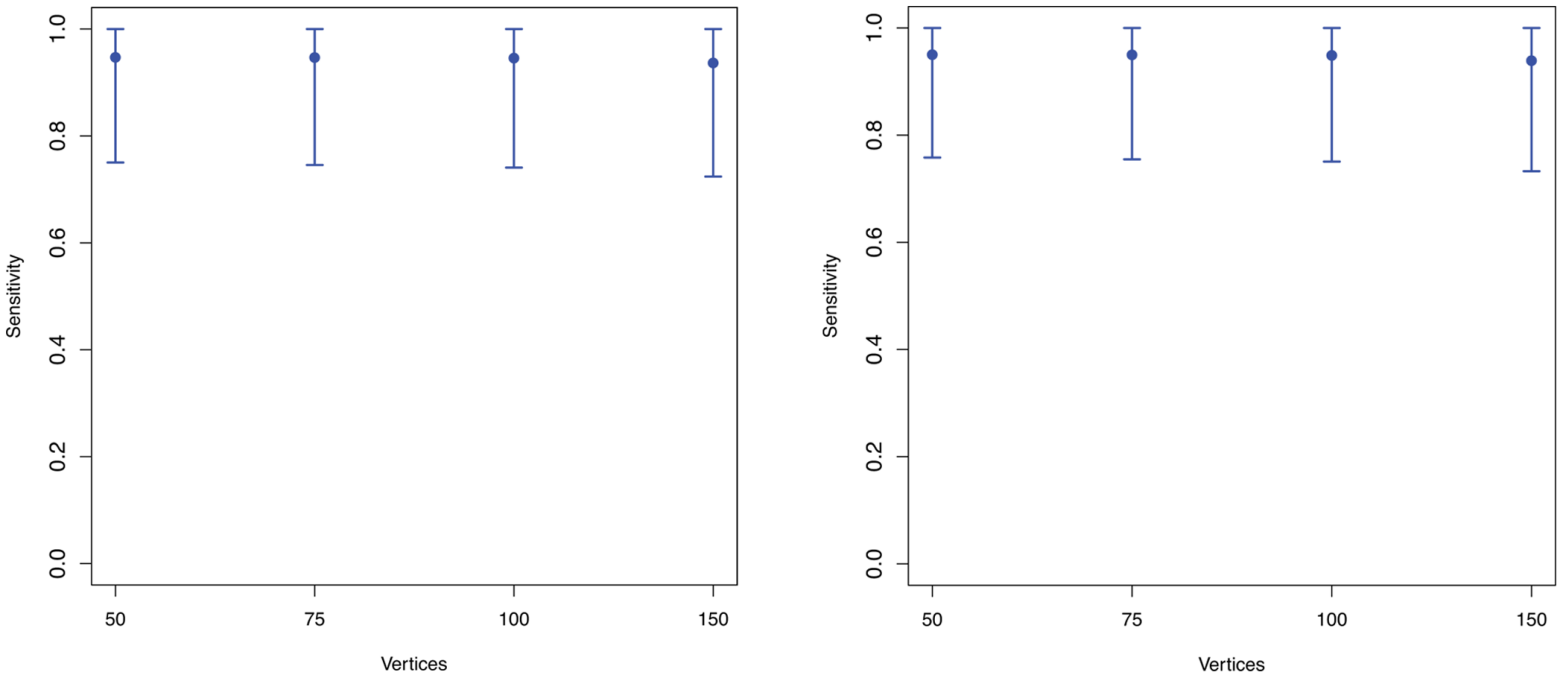
The means of the sensitivity values 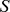 (see Equation 10) vs. the vertex sizes using the superindices 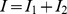 (Left) and 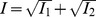
(Right).

**Figure 2:**
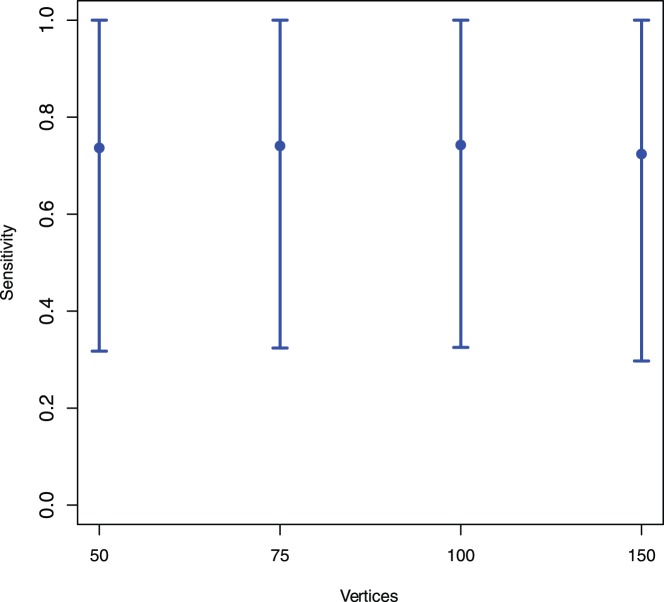
The means of the sensitivity values 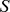 vs. the vertex sizes of the generated random graphs using the individual indices from Table 2 only.

For the statistical analysis see Figures 1 and 2. Samples of random (Erdös-Rényi) graphs have been generated using the R-library igraph [53] for 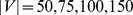. More precisely, we have generated 50 random graphs for each of the edge sizes 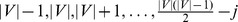. The parameter 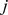 denotes the bound on the size of the random sample dictated by the computational algorithm. The procedure we used is detailed in the following algorithm.

### Algorithm 1

1. *Generate a connected random graph possessing 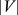 vertices and 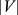-1 edges.*
2. *Add edges randomly between non-adjacent vertices to obtain edge sizes 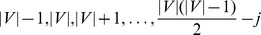.*
3. *Check each generated random graph for isomorphism with previously generated graphs. If the newly generated graph is not isomorphic to any of the previously generated graphs, we add this graph to the list, and return to step 1.*

Performing the computation in Algorithm 1, we obtain complete random samples for 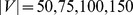. For the sake of completeness, we also give the sizes of the random samples generated:

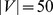
 and 
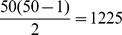
. By choosing 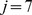, we generated random graphs with 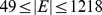. Hence, we obtain 58500 random graphs in total.

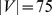
 and 
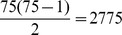
. By choosing 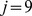, we generated random graphs with 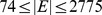. Hence, we obtain 134650 random graphs in total.

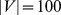
 and 
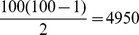
. By choosing 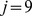, we generated random graphs with 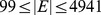. Hence, we obtain 242150 random graphs in total.

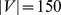
 and 
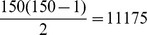
. By choosing 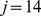, we generated random graphs with 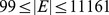. Hence, we obtain 550650 random graphs in total.

In order to calculate the superindices, we computed all possible (pairwise) combinations of the descriptors given in Table 2. To calculate the mean sensitivity 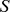 for each descriptor combination, we bootstrapped the samples 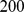-times without replacement. Finally, the mean values of all sensitivity values for superindices 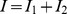 and 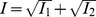 together with their variances are shown by Figures 1 and 2. The mean values are quite *stable*. Thus, there is little dependency between the mean sensitivity and the number of vertices of the generated random graphs. In particular, we see that the mean value detoriates slightly for 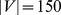. In short, Figure 1 strongly supports the hypothesis that the computed superindices have high discrimination power for graphs of increasing size and the values are quite stable. Indeed, *stability* could be defined here by the degree of the dependency between the mean sensitivity values and the number of vertices. Note that the analysis whose results are shown in Figure 1 was computationally demanding due to the combinatorial explosion of cases. Hence, to repeat the analysis for much larger (i.e., 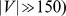) may not be feasible.

**Table 2:** Individual QuACN-descriptors.

eigenvaladj	balabanlike2
eigenvaldistance	bertz
eigenvaldistancepath	bonchev2
eigenvalextadj	bonchev3
eigenvalrandomwalk	compactness
eigenvalvertconnect	complexityIndexB
eigenvalaugement	eigenvallaplace
eigenvalweightedexp	graphDistanceComplexity
eigenvalweightedlin	mediumArticulation
laplacianEnergy	modifiedZagreb
laplacianEstrada	offdiagonal
spectralRadius	symmetryIndex
infotheoexp1	graphVertexComplexity
infotheoexp2	harary
infotheoexp3	hyperDistancePathIndex
infotheoexp4	meanDistanceDeviation
infotheolin1	variableZagreb
infotheolin2	vertexDegree
infotheolin3	normalizedEdgeComplexity
infotheoquad1	radialCentric
infotheoquad2	randic
infotheoquad3	topologicalinfocontent
augmentedZagreb	wiener
graphIndexComplexity	zagreb1
minBalabanID	zagreb2
balabanJ	narumiKatayama
balabanlike1	

In contrast to the superindices, the results in Figure 2 show that the discrimination power of the individual descriptors listed in Table 2 is worse for larger graphs. This is indicated by the mean sensitivity values which are much lower than the ones shown in Figure 1. This demonstrates that superindices 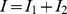 and 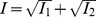 have a much better discrimination power on the generated random graphs. A reason for this is that the superindices seem to capture structural information more meaningfully than the individual ones. This seems to be clear (for the used graph class) as multiple descriptors capture several different aspects of structural information which may complement each other and, thus, provide a (super) index with improved discrimination power.

**Figure 3:**
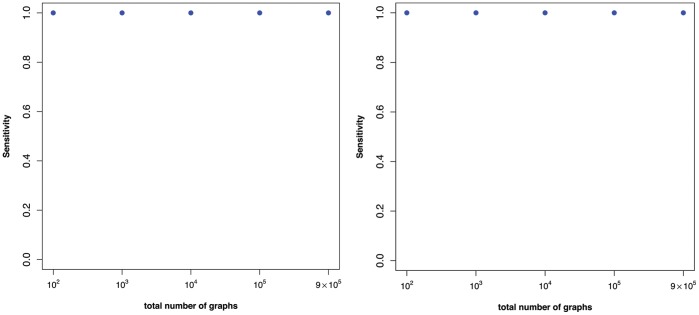
The means of the sensitivity values 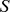 vs. the total number of randomly generated graphs 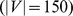 using the superindex 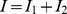. To calculate the superindex, we used all combinations of eigenvalue-based descriptors (Left) and eigenvalue-based and information-theoretic descriptors (Right), see Table 3.

**Figure 4:**
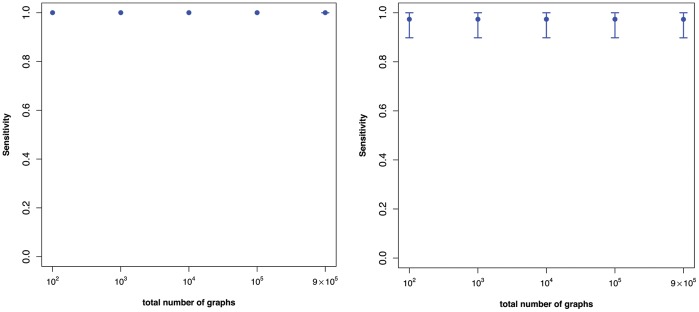
The means of the sensitivity values 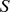 vs. the total number of randomly generated graphs 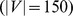 using the superindex 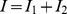. To calculate the superindex, we used all combinations of eigenvalue-based and distance-based descriptors (Left) and eigenvalue-based and degree-based descriptors (Right), see Table 3.

The results in Figures 1 and 2 summarize the uniqueness of some superindices as a function of the size of randomly generated graphs. We next consider the relationship between uniqueness (measured by 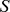) and graph size. The results are shown in Figure 3, 4, 5. Earlier work by Dehmer et al. [28] on superindices restricted the component individual indices to information-theoretic measures. In the present study, we aim to examine the dependency between the uniqueness of the superindex 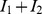 using certain descriptor categories applied to generated random graphs of fixed size (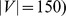. The categories included eigenvalue-based, information-theoretic, distance-based and degree-based descriptors. The descriptors in the categories are listed in Table 3. In order to calculate the mean sensitivity using the descriptors of the above mentioned categories, we bootstrapped the descriptor values 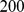 times without replacement for each combination to determine 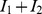 of randomly generated graphs (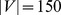). The sample sizes are 100, 1000, 10000, 100000, 900000.

**Figure 5:**
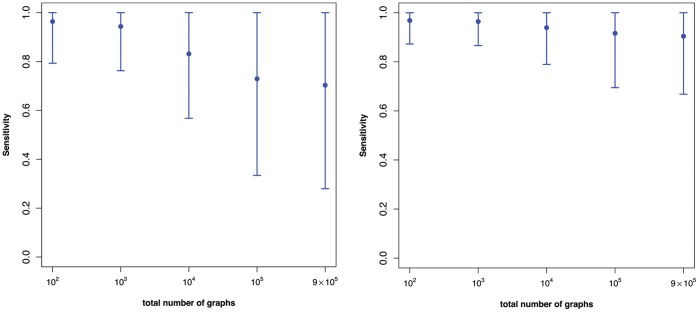
The means of the sensitivity values 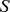 vs. the total number of randomly generated graphs 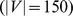 using the superindex 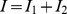. To calculate the superindex, we used all combinations of distance-based descriptors (Left) and distance-based and degree-based descriptors (Right), see Table 3.

**Table 3:** Categories of QuACN-descriptors.

Eigenvalue-based descriptors
eigenvaladj, eigenvallaplace, eigenvaldistance, eigenvaldistancepath, eigenvalaugement, eigenvalextadj, eigenvalvertconnect, eigenvalrandomwalk, eigenvalweightedlin, eigenvalweightedexp, laplacianEnergy, laplacianEstrada, spectralRadius
**Information-theoretic descriptors**
infotheolin1, infotheolin2, infotheoquad1, infotheoquad2, infotheoexp1, infotheoexp2, infotheoexp3, infotheolin3, infotheoquad3, infotheoexp4
**Distance-based descriptors**
balabanJ, balabanlike1, balabanlike2, bertz, bonchev2, bonchev3, compactness, complexityIndexB, wiener, harary, radialCentric, meanDistanceDeviation, hyperDistancePathIndex, topologicalinfocontent, graphVertexComplexity, graphDistanceComplexity, symmetryIndex, productofrowsums
**Degree-based descriptors**
augmentedZagreb, randic, zagreb1, zagreb2, vertexDegree, modifiedZagreb, narumiKatayama, offdiagonal, variableZagreb

Figures 3, 4, 5 shows the impact of the underlying category on the above mentioned dependency. From Figure 3 we see that there is nearly no dependency between 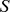 and the sample size. A plausible reason for this is the high uniqueness of the underlying individual descriptors of the categories employed, namely, (left) eigenvalue-based descriptors and (right) eigenvalue-based and information-theoretic descriptors (see Table 4). Figure 4 shows a similar result but there is a slight detoriation of uniqueness for the degree-based descriptors used calculate the superindex. This seems plausible as many degree-based measures possess little discrimination power, e.g., see [31]. The left hand side of Figure 5 shows the dependency plot by using the (pure) category of distance-based measures (see Table 3). In particular, the variances are very high and the mean sensitivity values detoriate substantially as the sample size increases. Again, this can be understood by the low uniqueness of various distance-based graph measures (see Table 4). The right hand side of Figure 5 shows that this effect is eased for a (mixed) category of descriptors - distance-based and degree-based descriptors in the present case. In summary, we see that the uniqueness of the superindex does not depend much on the sample size when the component descriptors are relatively unique. In our study, this applies to the eigenvalue-based and information-theoretic descriptors. It is not surprising that we obtained very similar results by using the superindex 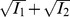.

## Summary and Conclusion

In the foregoing we examined the discrimination power of structural superindices composed of two or more individual measures (or descriptors) defined on graphs. Our results show that superindices generally have greater discrimination power than individual descriptors. The initial analysis of the superindices was performed the collection of graphs on nine vertices. In addition, we examined the relative performance of superindices on randomly generated connected graphs on 50, 75, 100, and 150 vertices, respectively. The findings show that the superindices perform consistently over these different sized graphs, whereas individual descriptors exhibit declining performance. We conjecture that this superior performance of superindices is attributable to their taking account of multiple structural features of a graph, rather than the single feature captured by individual descriptors. Further research is needed to account for the differences in performance between different superindices, and between superindices and individual descriptors.
