# Correction: The Discrimination Power of Structural SuperIndices

**DOI:** 10.1371/annotation/792f2983-0889-432a-b047-e1c9577dcc7e

**Published:** 2013-08-02

**Authors:** Matthias Dehmer, Abbe Mowshowitz

Institutional affiliation 1 for author Matthias Dehmer is incorrect. The correct affiliation is: 1. UMIT, Institute for Bioinformatics and Translational Research, Eduard Wallnoefer Zentrum 1, Hall in Tyrol, Austria. 

